# Development of Novel ^11^C-Labeled Selective Orexin-2 Receptor Radioligands for Positron Emission Tomography Imaging

**DOI:** 10.1021/acsmedchemlett.3c00320

**Published:** 2023-09-26

**Authors:** Jian Rong, Tomoteru Yamasaki, Yinlong Li, Katsushi Kumata, Chunyu Zhao, Achi Haider, Jiahui Chen, Zhiwei Xiao, Masayuki Fujinaga, Kuan Hu, Wakana Mori, Yiding Zhang, Lin Xie, Xin Zhou, Thomas L. Collier, Ming-Rong Zhang, Steven Liang

**Affiliations:** † Department of Radiology and Imaging Sciences, Emory University, Atlanta, Georgia 30322, United States; ‡ Division of Nuclear Medicine and Molecular Imaging, Massachusetts General Hospital and Department of Radiology, Harvard Medical School, Boston, Massachusetts 02114, United States; § Department of Advanced Nuclear Medicine Sciences, Institute for Quantum Medical Sciences, National Institutes for Quantum Science and Technology, Chiba 263-8555, Japan

**Keywords:** Orexin-2, PET, antagonist, C-11

## Abstract

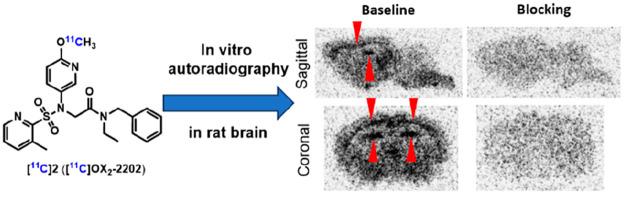

Orexin 2 receptors (OX_2_R) represent a vital subtype of orexin receptors intricately involved in the regulation of wakefulness, arousal, and sleep–wake cycles. Despite their importance, there are currently no positron emission tomography (PET) tracers available for imaging the OX_2_R in vivo. Herein, we report [^11^C]**1** ([^11^C]OX_2_-2201) and [^11^C]**2** ([^11^C]OX_2_-2202) as novel PET ligands. Both compounds **1** (*K*_i_ = 3.6 nM) and **2** (*K*_i_ = 2.2 nM) have excellent binding affinity activities toward OX_2_R and target selectivity (OX_2_/OX_1_ > 600 folds). *In vitro* autoradiography in the rat brain suggested good to excellent *in vitro* binding specificity for [^11^C]**1** and [^11^C]**2**. PET imaging in rat brains indicated that the low brain uptake of [^11^C]**2** may be due to P-glycoprotein and/or breast cancer resistance protein efflux interaction and/or low passive permeability. Continuous effort in medicinal chemistry optimization is necessary to improve the brain permeability of this scaffold.

In 1998, the orexin system (hypocretin) was first reported^1,2^ to be responsible for a range of physiological functions such as controlling food intake, energy homeostasis, and sleep–wake regulation.^3−5^ The orexin system consists of two G-protein-coupled receptors (GPCRs) named orexin 1 receptor (OX_1_R) and orexin 2 receptor (OX_2_R), and the corresponding coupled endogenous neuropeptide ligands, orexin-A (a 33 amino acid peptide) and orexin-B (a 28 amino acid peptide).^6^ The OX_1_R and OX_2_R have distinct expression patterns throughout the central nervous system (CNS), and OX_1_R is mainly located in the locus coeruleus, whereas OX_2_R is distributed in the cortex, septal nuclei, lateral hypothalamus, and hippocampus, indicating that the orexin receptor subtypes may possess unique physiological and psychiatric roles via the differentiated neuronal mechanism.^7,8^ In general, orexin-A is a nonselective neuropeptide binding both OX_1_R and OX_2_R with similar affinities, while orexin-B has an improved selectivity toward OX_2_R against OX_1_R (>10 folds).^9^ Previous studies revealed that OX_1_R/OX_2_R knockout (KO) mice display a severe narcoleptic phenotype.^10^ Compared to OX_1_R/OX_2_R KO mice, OX_2_R KO mice exhibit a relatively milder narcoleptic phenotype, and OX_1_R KO mice show no appreciable narcoleptic phenotype.^11^ Furthermore, the disruption of the OX_2_R gene was identified to be responsible for narcolepsy in a well-established canine model.^12^ Although researchers still have divergent ideas on the signaling pathways of OX_1_R/OX_2_R-derived disorders, these findings present evidence that OX_2_R may be the predominant modulator for the maintenance of sleep–wake cycles, and the regulation of OX_2_R-involved signaling may be sufficient for the treatment of narcolepsy/cataplexy.^13,14^

Notably, intracerebroventricular infusion of orexins in rats has been disclosed to improve behavioral activity and attenuate slow-wave sleep.^15^ Further clinical evidence has demonstrated that human narcoleptic patients have declined orexin peptide levels as well as weakened orexinergic neurons, indicating the therapeutic potential for insomnia-related disorders through blocking orexin signaling with OX_1_R/OX_2_R antagonists.^16,17^ Numerous drug discovery efforts have been devoted to the development of orexin antagonists for insomnia therapy.^18,19^ Recently, a number of orexin receptor antagonists have progressed into clinical trials. For example, Merck’s dual orexin receptor antagonists (DORAs) named suvorexant the first marketed orexin antagonist for primary insomnia treatment, which was approved by the U.S. Food and Drug Administration (FDA) in 2014 (see Supporting Information, Figure S1, a list of orexin 2 receptor antagonists).^20^ Furthermore, the development of novel and selective orexin-2 antagonists is considered as a promising topic of research in sleep disorder therapy, since evidence continues to validate that the selective inhibition of OX_2_R alone is more adequate for sleep promotion. In recent years, several pharmaceutical molecules have been designed and evaluated as selective orexin-2 antagonists for the treatment of insomnia (Figure S1).^21−23^ However, only a few of these molecules have entered clinical trials, and there is no selective orexin-2 antagonist on the market to date. As a noninvasive nuclear imaging technique, positron emission tomography (PET) represents a powerful tool for quantitative measurement of various biological processes *in vivo*.^24−26^ To investigate the distribution of the OX_2_R and the evaluation of the OX_2_R-targeted neurotherapeutics, there have been continuous efforts in the development of radiolabeled orexin-2 antagonists in the past several years. For instance, [^11^C]EMPA,^27^ [^11^C]BBAC,^28^ [^11^C]CW4,^29^ [^11^C]FFMMCC,^30^ [^11^C]MK-1064,^31^ and [^18^F]DAN-1^32^ have been developed as potential PET radioligands for imaging of OX_2_R (Figure 1). Unfortunately, most of these radiolabeled tracers display insufficient brain uptake or relatively low binding affinity for OX_2_R, which limits further translation in clinical research studies. Therefore, it represents an urgent and unmet need to develop novel potent and selective probes for OX_2_R imaging with high binding affinity, high selectivity, favorable brain uptake, and washout.

**Figure 1:**
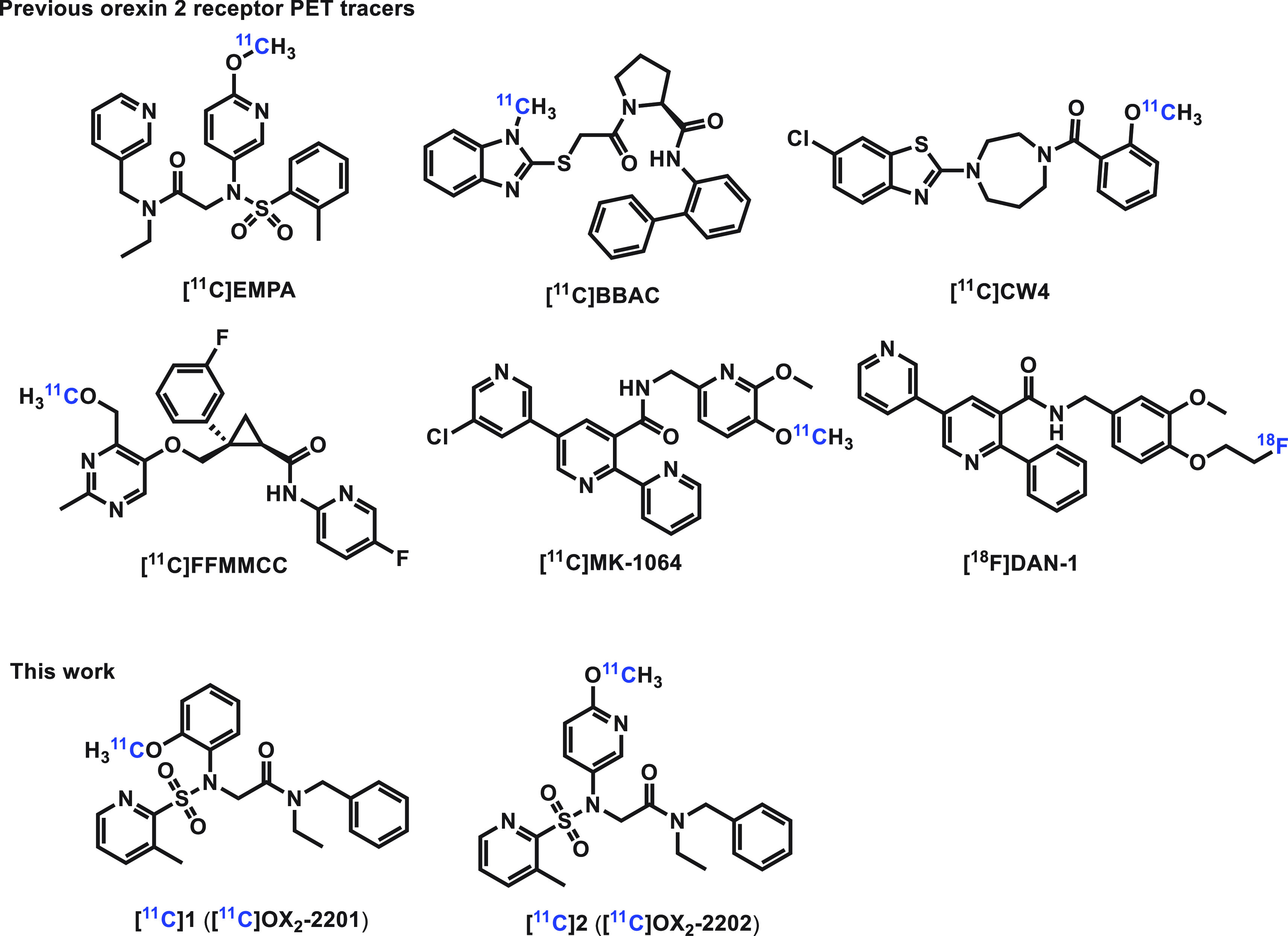
Representative orexin 2 receptor PET tracers.

With the goal of developing a potent and selective OX_2_R PET tracer, we started with the EMPA scaffold, which has already demonstrated excellent antagonist potency (IC_50_ = 2 nM).^33^ The in silico permeability across the BBB (log BB = −1.05) of EMPA was predicted with low possibility by ACD/Percepta (software used to predict the most common physicochemical, pharmacokinetic, and toxicology properties; ACD/Laboratories, Toronto, ON, Canada, https://www.acdlabs.com/products/percepta). We then modified the structure of EMPA and designed two new analogs **1** and **2** with higher log BB and log *P* values (log BB = −0.01 and −0.20, and log *P* = 3.06 and 2.29 for **1** and **2**, respectively) predicted by ACD/Percepta, aiming for an improved BBB penetration. The topological polar surface areas (tPSA’s) for EMPA, **1**, and **2** were predicted by ChemDraw 22.2, indicating the appropriate physicochemical properties for them. Herein, we report the radiosynthesis and preliminary evaluation of [^11^C]**1** ([^11^C]OX_2_-2201) and [^11^C]**2** ([^11^C]OX_2_-2202) as novel and potential PET ligands for imaging the orexin 2 receptor (Figure 1).

With compounds **1** and **2** as the molecules of interest, we performed an efficient multistep synthesis of standards and the corresponding phenolic precursors for ^11^C-labeling. The synthesis of compound **1** and its phenolic precursor **7** was summarized in Figure 2. Sulfonamide **4** was initially attempted from cross-coupling reactions of 3-methylpyridine-2-sulfonamide and 1-iodo-2-methoxybenzene (path A), but it was unsuccessful. Then, we rerouted with path B, and sulfonamide **4** was obtained from the reaction of amine **3** with sulfonyl chloride in 21% yield. The following nucleophilic substitution was successfully realized with methyl 2-bromoacetate, leading to key intermediate **5** in 57% yield. Hydrolysis of compound **5** under basic conditions gave carboxylic acid **6**, which was directly used in the condensation reactions with *N*-benzylethanamine without further purification, to generate compound **1** with 29% yield. The phenolic precursor **7** was prepared from the demethylation of compound **1** in the presence of boron tribromide in 41% yield.

**Figure 2:**
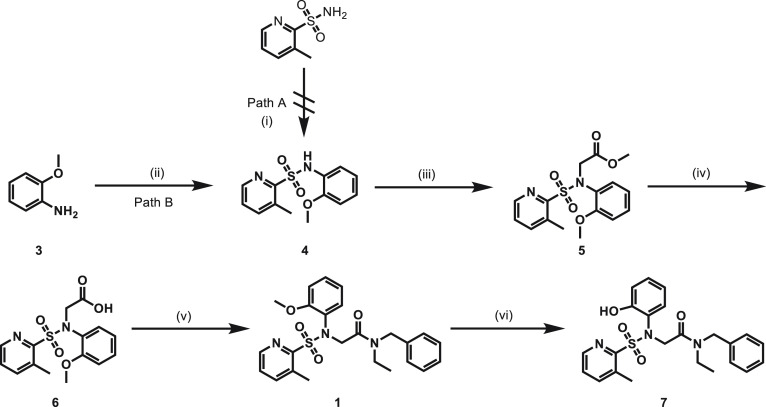
Synthesis of orexin 2 receptor radioligand **1** and its precursor **7**. (i) 1-Iodo-2-methoxybenzene, CuI, Cs_2_CO_3_, DMF, 130 °C, 12 h, 0%; (ii) 3-methylpyridine-2-sulfonyl chloride, CH_2_Cl_2_, 0 °C to r.t., 3 h, 21%; (iii) methyl 2-bromoacetate, K_2_CO_3_, DMF, 25 °C, 12 h, 57%; (iv) NaOH, MeOH/H_2_O/THF, 60 °C, 12 h, 62%; (v) *N*-benzylethanamine, HATU, *i*Pr_2_NEt, CH_2_Cl_2_, r.t., 12 h, 29%; (vi) BBr_3_, CH_2_Cl_2_, −10 °C, 5 min, 41%. DMF = dimethylformamide, HATU = 1-[Bis(dimethylamino)methylene]-1*H*-1,2,3-triazolo[4,5-*b*]pyridinium 3-oxide hexafluorophosphate.

The synthesis of compound **2** and its phenolic precursor **12** was summarized in Figure 3. The synthesis of sulfonamide **9** was initially designed from the reaction of 6-methoxypyridin-3-amine and pyridine-2-sulfonyl chloride (path A), but it was not successful. Alternatively, sulfonamide **9** was then obtained from the cross-coupling reaction of 3-methylpyridine-2-sulfonamide **8** and 5-iodo-2-methoxypyridine in 60% yield (path B). The following nucleophilic substitution was successfully realized with methyl 2-bromoacetate, leading to key intermediate **10** in 99% yield. Hydrolysis of compound **10** under basic conditions gave carboxylic acid **11**, which was directly used in the condensation reactions with *N*-benzylethanamine without further purification, to achieve compound **2** in 40% yield. The phenolic precursor **12** was prepared from the demethylation of compound **2** in the presence of hydrogen bromide in 45% yield.

**Figure 3:**
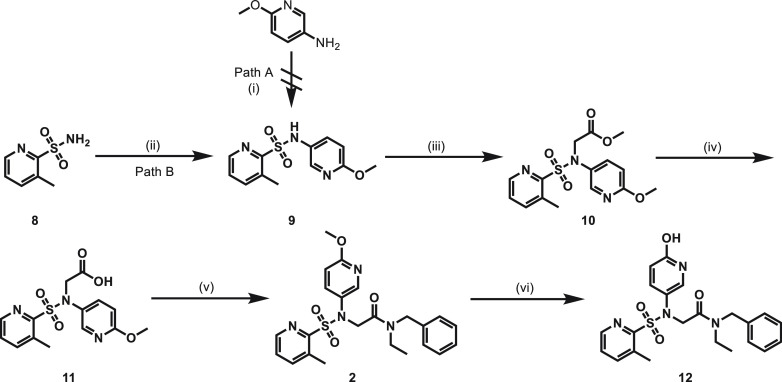
Synthesis of orexin 2 receptor radioligand **2** and its precursor **12**. (i) Pyridine-2-sulfonyl chloride, pyridine, 0 °C to rt, 1 h, 0%; (ii) 5-iodo-2-methoxypyridine, Cs_2_CO_3_, CuI, DMF, 150 °C, microwave, 30 min, 60%; (iii) methyl 2-bromoacetate, *t*BuOK, DMSO, 60 °C, 12 h, 99%; (iv) NaOH, MeOH/H_2_O, 60 °C, 3 h, 85%; (v) *N*-benzylethanamine, HATU, *i*Pr_2_NEt, CH_2_Cl_2_, rt, 12 h, 40%; (vi) HBr/HOAc, 60 °C, 12 h, 45%. DMF = dimethylformamide, HATU = 1-[Bis(dimethylamino)methylene]-1*H*-1,2,3-triazolo[4, 5-*b*]pyridinium 3-oxide hexafluorophosphate.

To determine the binding affinities of compounds **1** and **2** toward human orexin 2 receptor, we performed the radioligand ([^3^H]EMPA) competition assays. As shown in Figure 4, compound **1** had an IC_50_ value of 7.1 nM and a *K*_i_ value of 3.6 nM. Compound **2** had a better potency and binding affinity toward OX_2_ (IC_50_ = 4.4 nM and *K*_i_ = 2.2 nM). In a selected list of CYP450-mediated drug metabolism panels, low inhibition (IC_50_ > 5 μM) was observed for CYP1A2, CYP2C9, CYP2C19, CYP2D6, CYP3A4, and CYP19A for both compounds **1** and **2** (Figure S2). Both compounds **1** and **2** displayed low hERG inhibition (IC_50_ > 100 μM; Figure S2). Furthermore, we investigated the off-target binding of compounds **1** and **2** in vitro toward more than 50 major transporters, ion channels, and GPCR enzymes in the CNS (supported by the NIMH PDSP). As shown in Figures S3 and S4, no significant off-target binding (all <50% inhibition) toward 54 CNS targets was observed for both compounds **1** and **2** at a concentration of 10 μM, except the peripheral benzodiazepine receptor for compound **1** (*K*_i_ = 253 nM).

**Figure 4:**
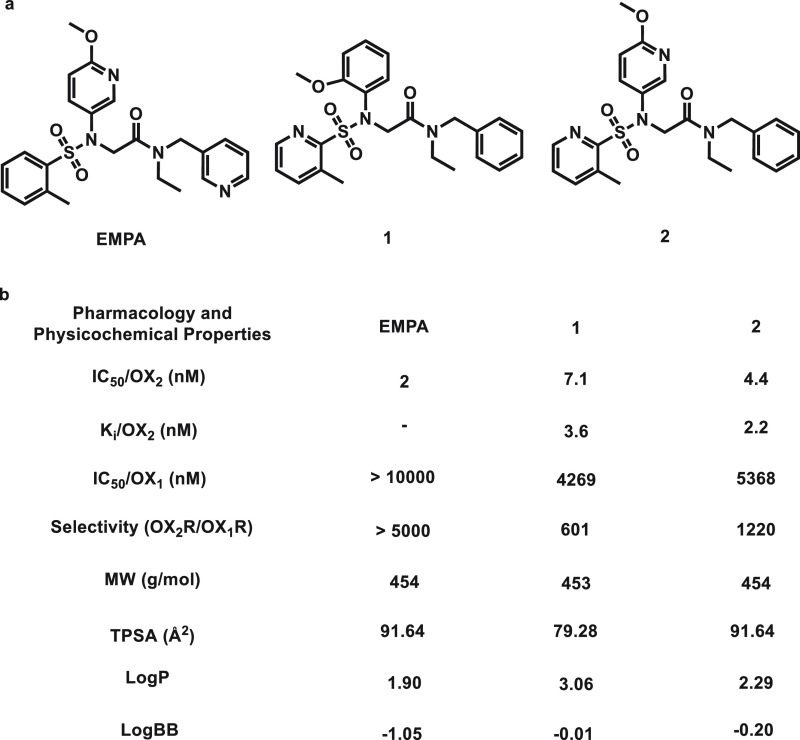
OX_2_ radioligands EMPA, **1**, and **2**. (a) Chemical structures of EMPA, **1**, and **2**. (b) Representative pharmacological and ADME properties of EMPA, **1**, and **2**.

Based on the favorable in vitro pharmacological and ADME properties, compounds **1** and **2** were labeled with carbon-11 and evaluated as OX_2_ PET radioligands. The radiosynthesis of [^11^C]**1** and [^11^C]**2** was conducted by ^11^C-methylation^34^ of phenolic precursors with [^11^C]CH_3_I, utilizing NaOH or Cs_2_CO_3_ as the base in DMF at 80 °C for 5 min (Figure 5). The reaction mixtures were then diluted with the HPLC buffer and purified by a semipreparative reverse HPLC to give [^11^C]**1** and [^11^C]**2** in 71% and 20% radiochemical yields (decay-corrected) with the molar activities of 210 GBq/μmol and 185 GBq/μmol, respectively. The radiochemical purities of both tracers were greater than 99%, and both [^11^C]**1** and [^11^C]**2** were stable in saline within 90 min.

**Figure 5:**
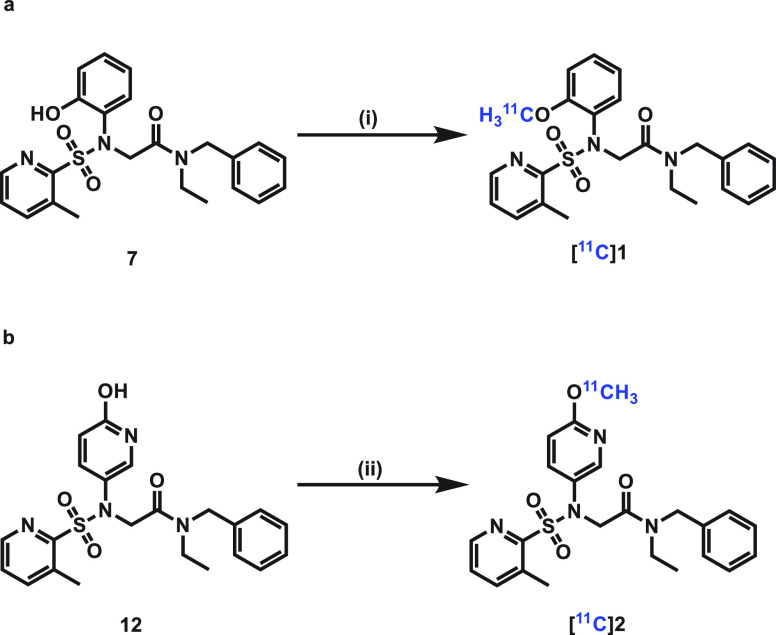
Radiosynthesis of [^11^C]**1** and [^11^C]**2**. (a) Radiosynthesis of [^11^C]**1**, (i) [^11^C]CH_3_I, NaOH, and DMF, 80 °C, 5 min; 71% decay-corrected RCY. (b) Radiosynthesis of [^11^C]**2**, (ii) [^11^C]CH_3_I, Cs_2_CO_3_, and DMF, 80 °C, 5 min; 20% decay-corrected RCY.

With the ligands [^11^C]**1** and [^11^C]**2** in hand, we subsequently conducted *in vitro* autoradiography studies using rat brain sections to investigate the binding specificity of [^11^C]**1** and [^11^C]**2** toward orexin 2 receptors. As shown in Figure 6, in the baseline experiments with [^11^C]**1** and [^11^C]**2**, relatively high radioactivity accumulation was observed in cortical layer 6 (L6), cornu ammonis 3 (CA3), the central bed nucleus (CM), and medial hypothalamus (MH). Blocking studies with compounds **1** (10 μM), **2** (10 μM), and EMPA (10 μM) remarkably diminished the radioactivity in the OX_2_-rich brain regions. These results suggested good to excellent *in vitro* binding specificity for both [^11^C]**1** and [^11^C]**2**, in which ligand [^11^C]**2** had superior specific binding in the rat brain. Besides our blocking studies using EMPA analogs, it is imperative that a structurally distinct OX_2_R blocker is applied in future studies to fully validate the binding specificity in vitro.

**Figure 6:**
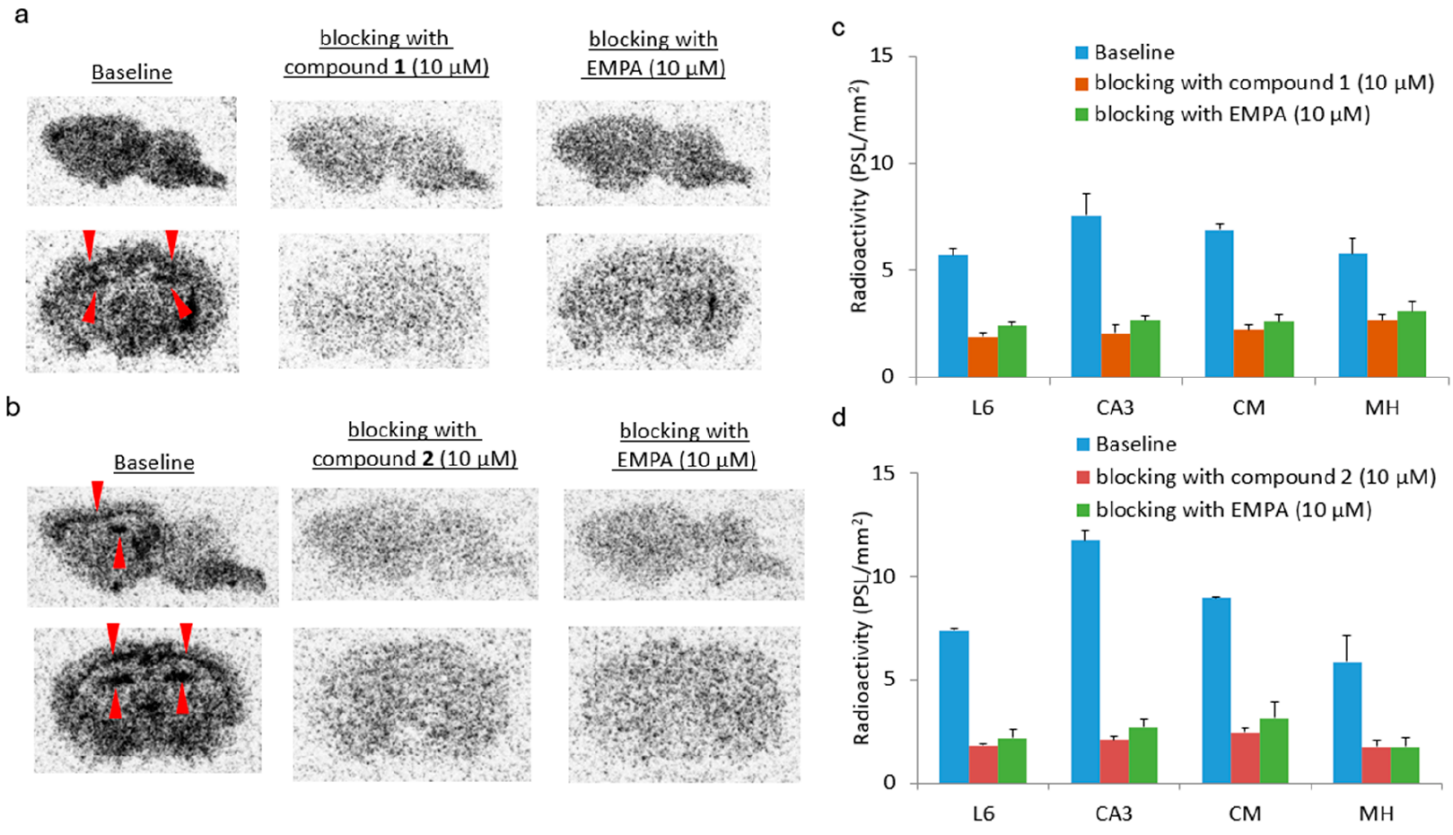
In vitro autoradiography of [^11^C]**1** and [^11^C]**2** in rat brains. (a) Representative images for baseline and blocking autoradiography studies (compound **1**, 10 μM or EMPA, 10 μM) with [^11^C]**1**. (b) Representative images for baseline and blocking autoradiography studies (compound **2**, 10 μM or EMPA, 10 μM) with [^11^C]**2**. (c) Quantification of autoradiography studies with [^11^C]**1** and (d) quantification of autoradiography studies with [^11^C]**2**. L6 = cortical layer 6, CA3 = cornu ammonis 3, CM = central bed nucleus, MH = medial hypothalamus. All data were referred to as mean ± SD, *n* ≥ 3.

Encouraged by the autoradiography results of ligand [^11^C]**2**, dynamic PET imaging of [^11^C]**2** was then performed in Sprague–Dawley (SD) rats for 60 min. As depicted in Figure 7, ligand [^11^C]**2** had poor brain uptake, and the highest radioactivity levels in the hippocampus and cerebellum were around 0.4 SUV (2 min postadministration of tracer), followed by washout to 0.2 SUV. Preadministration of OX_2_ antagonist, EMPA (1 mg/kg, i.v.), prior to tracer injection, did not remarkably reduce the bound radioactivity levels in the hippocampus and cerebellum (Figure S5). To investigate the effect of P-glycoprotein (Pgp) and breast cancer resistance protein (BCRP) efflux on brain uptake, we subsequently performed PET imaging of [^11^C]**2** in SD rats with Elacridar (pretreatment), an inhibitor of Pgp and BCRP. Specifically, the SD rats were treated with Elacridar (3 mg/kg) 20 min before injection of the [^11^C]**2**.^35^ The highest radioactivity levels in the hippocampus and cerebellum increased to 0.9 SUV, followed by a quick washout to 0.3 SUV. Although the ligand [^11^C]**2** may be the substrate of Pgp and/or BCRP, low passive permeability could be another confounding factor for the relatively low SUV in the brain.

**Figure 7:**
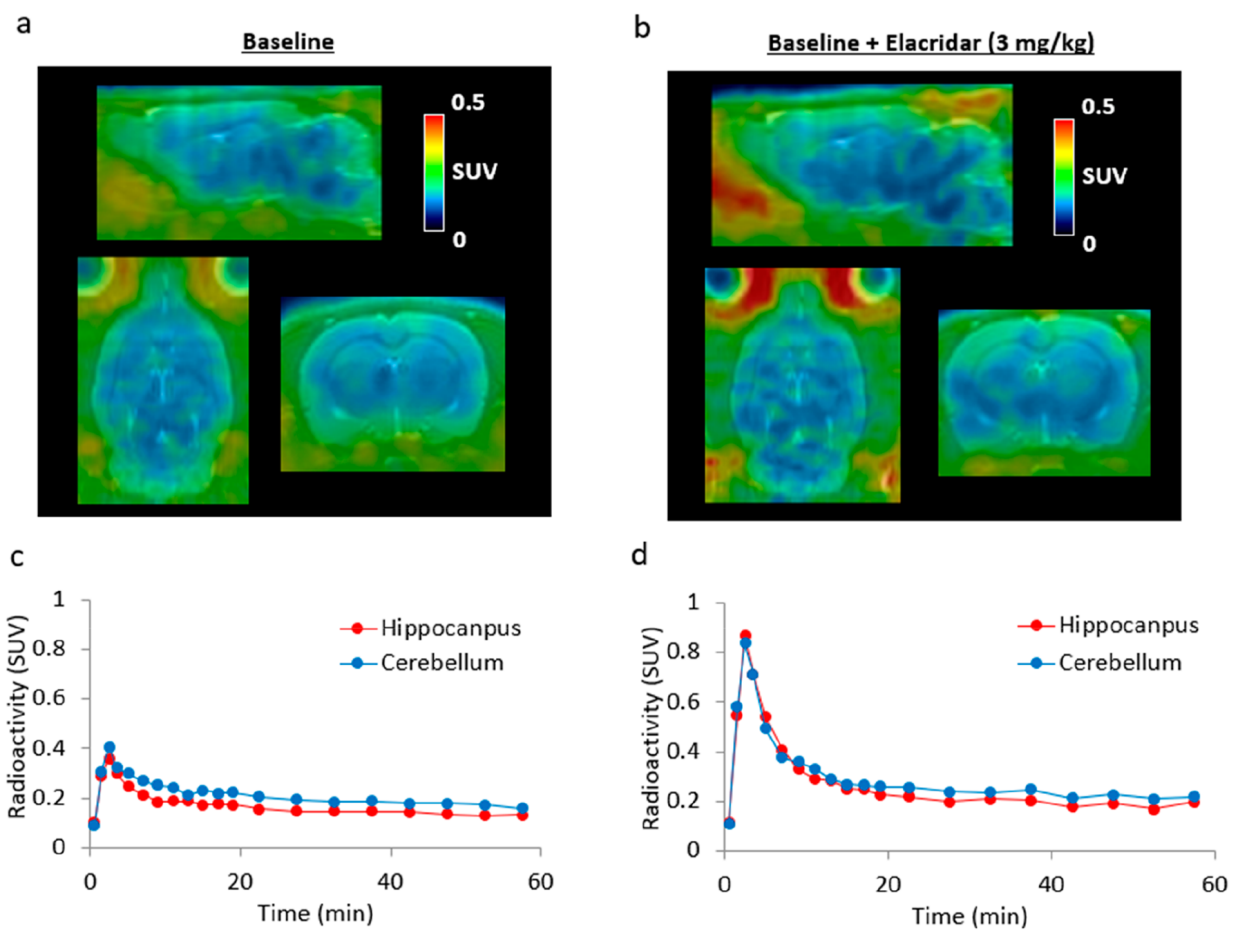
PET studies of [^11^C]**2** in rat brains. (a and b) Representative summed PET images (0–60 min) of [^11^C]**2** under baseline and blocking conditions (Elacridar, 3 mg/kg); (c and d) TACs of [^11^C]**2** in hippocampus and cerebellum.

In conclusion, we have developed two novel OX_2_R PET ligands, compounds **1** and **2**. Pharmacological evaluation identified compounds **1** and **2** with high binding affinity (*K*_i_ = 3.6 nM and 2.2 nM, respectively) and excellent target selectivity (OX_2_/OX_1_ > 600 folds for both **1** and **2**). The PET ligands [^11^C]**1** ([^11^C]OX_2_-2201) and [^11^C]**2** ([^11^C]OX_2_-2202) were both prepared in high decay-corrected RCYs (71% and 20%, respectively) with high molar activities (210 and 185 GBq/μmol, respectively). Furthermore, evaluation of [^11^C]**1** and [^11^C]**2** by *in vitro* autoradiography in rat brains suggested good to excellent *in vitro* binding specificity, in which [^11^C]**2** demonstrated an improved specific binding toward OX_2_. PET imaging in rat brains suggested that the low brain uptake of [^11^C]**2** may be a mixed contribution of P-glycoprotein and/or breast cancer resistance protein efflux interaction and/or low passive permeability. Therefore, radioligands [^11^C]**1** and [^11^C]**2** are not suitable for imaging orexin 2 receptors in vivo, and continuous medicinal chemistry efforts are necessary to improve blood–brain barrier penetration of this scaffold.

## Supporting Information Available

Characterization of all new compounds and supplemental figures and tables (PDF)

## Abbreviations

CA3: Cornu Ammonis 3
CM: central bed nucleus
CNS: central nervous system
DORA: dual orexin receptor antagonist
FDA: Food and Drug Administration
GPCR: G protein-coupled receptor
OX_1_R: orexin 1 receptor
OX_2_R: Orexin 2 receptor
KO: knockout
L6: the cortical layer 6
MH: medial hypothalamus
PET: positron emission tomography
Pgp: P-glycoprotein
