# Supplementary material for: Development of Novel 11C-Labeled Selective Orexin-2 Receptor Radioligands for Positron Emission Tomography Imaging
Source: ACS Med Chem Lett. 2023 Sep 26;14(10):1419–26. doi: 10.1021/acsmedchemlett.3c00320 (PMC10577698; doi:10.1021/acsmedchemlett.3c00320)
Supplement: Supplementary file 1 — ml3c00320_si_001.pdf [file ml3c00320_si_001.pdf]

Development of Novel ^{11}C-labeled Selective Orexin-2 Receptor Radioligands for Positron Emission Tomography Imaging

Jian Rong[1,2,†], Tomoteru Yamasaki[3,†], Yinlong Li[1,2], Katsushi Kumata[3], Chunyu Zhao[1,2], Ahmed Haider[1,2], Jiahui Chen[1,2], Zhiwei Xiao[1,2], Masayuki Fujinaga[3], Kuan Hu[3], Wakana Mori[3], Yiding Zhang[3], Lin Xie[3], Xin Zhou[1], Thomas L. Collier[1,2], Ming-Rong Zhang[3,]*, and Steven Liang[1,2,]*

[1]Department of Radiology and Imaging Sciences, Emory University, Atlanta, GA 30322, USA

[2]Division of Nuclear Medicine and Molecular Imaging, Massachusetts General Hospital & Department of Radiology, Harvard Medical School, Boston, MA 02114, USA

[3]Department of Advanced Nuclear Medicine Sciences, Institute for Quantum Medical Sciences, National Institutes for Quantum Science and Technology, Chiba 263-8555, Japan

*Correspondence: steven.liang@emory.edu, zhang.ming-rong@qst.go.jp

[†]These two authors contributed equally to this work.

Table of contents

1. Supporting Figures S2
   Figure S1 Representative orexin 2 receptor antagonists. S2
   Figure S2 Pharmacological evaluation of compounds **1** and **2** against major CYP450 enzymes and hERG S2
   Figure S3 Off-target pharmacological evaluation of compound **1**. S3
   Figure S4 Off-target pharmacological evaluation of compound **2**. S3
   Figure S5 Representative PET images and time–activity curves of [^{11}C]**2** in blocking study in rat brains S4

2. Experimental section.. .S4
   2.1. General information S4
   2.2. Synthetic procedures and characterization data S5

2.3 Human OX_2 receptor binding assays and CYP enzymes and hERG inhibition assays....................S13
2.4. Radiolabeling of [^{11}C]**1** and [^{11}C]**2**....................S13
2.5. In vitro autoradiography....................S15
2.6. Small-animal PET imaging studies....................S15
2.7. HPLC radio-chromatograms of [^{11}C]**1** and [^{11}C]**2**....................S16
2.8. NMR spectra of key compounds **1** and **2**....................S19

1. Supporting figures

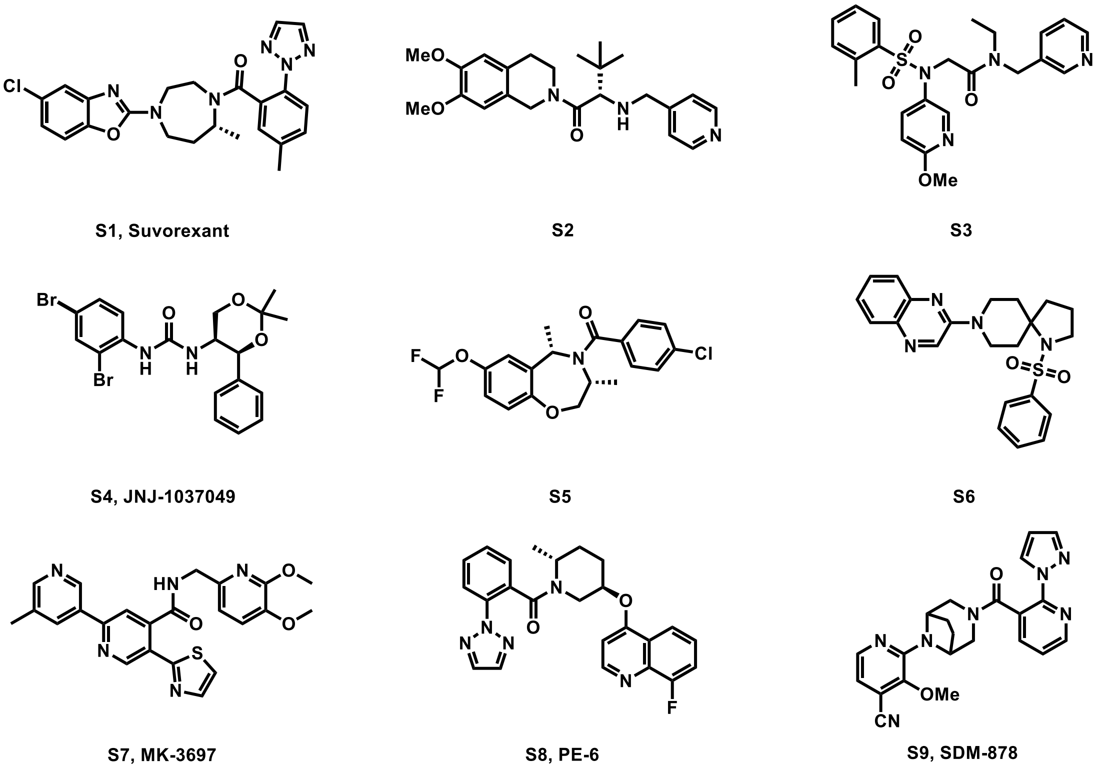

Figure S1. Representative orexin 2 receptor antagonists

Compounds	IC$_{50}$ towards CYP enzymes						hERG
	CYP1A2	CYP2C9	CYP2C19	CYP2D6	CYP3A4	CYP19A	
1	> 5 uM	> 5 uM	> 5 uM	> 5 uM	ND	> 5 uM	> 100 uM
2	> 5 uM	> 5 uM	> 5 uM	> 5 uM	> 5 uM	> 5 uM	> 100 uM

Figure S2. Pharmacological evaluation of compounds **1** and **2** against major CYP450 enzymes and hERG

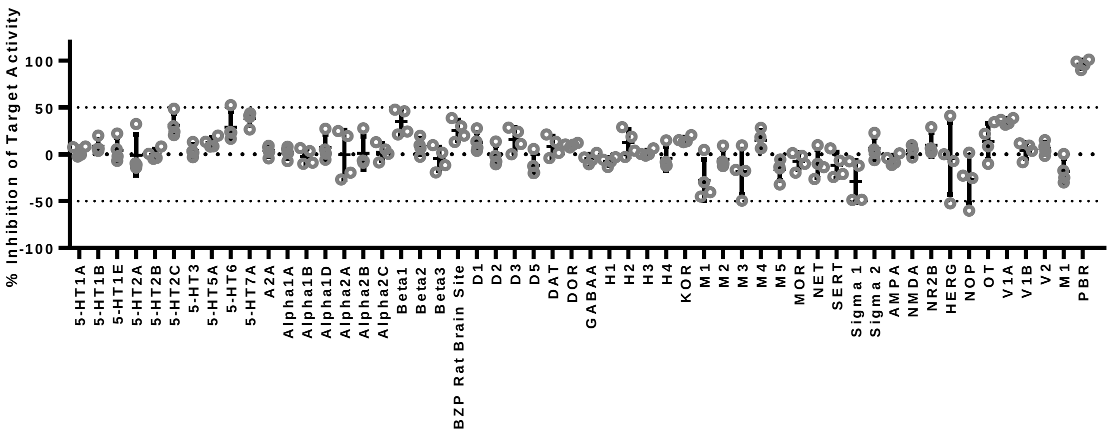

Figure S3. Off-target pharmacological evaluation of compound **1** at 10 μM against major CNS targets, including common GPCRs, enzymes, ion channels, and transporters: initial screening at 10 μM. All data are mean ± SD (n ≥ 4). No significant off-target binding (>50%) was observed at 10 μM compound testing concentration, except PBR (Ki = 253 nM).

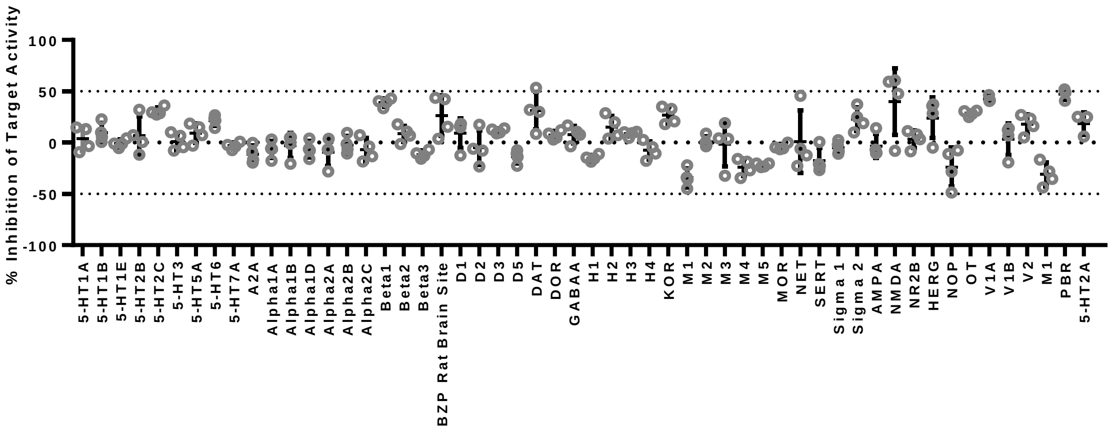

Figure S4. Off-target pharmacological evaluation of compound **2** at a concentration of 10 μM against major CNS targets, including common GPCRs, enzymes, ion channels, and transporters: initial screening at 10 μM. All data are mean ± SD (n ≥ 4). No significant off-target binding (>50%) was observed at 10 μM compound testing concentration.

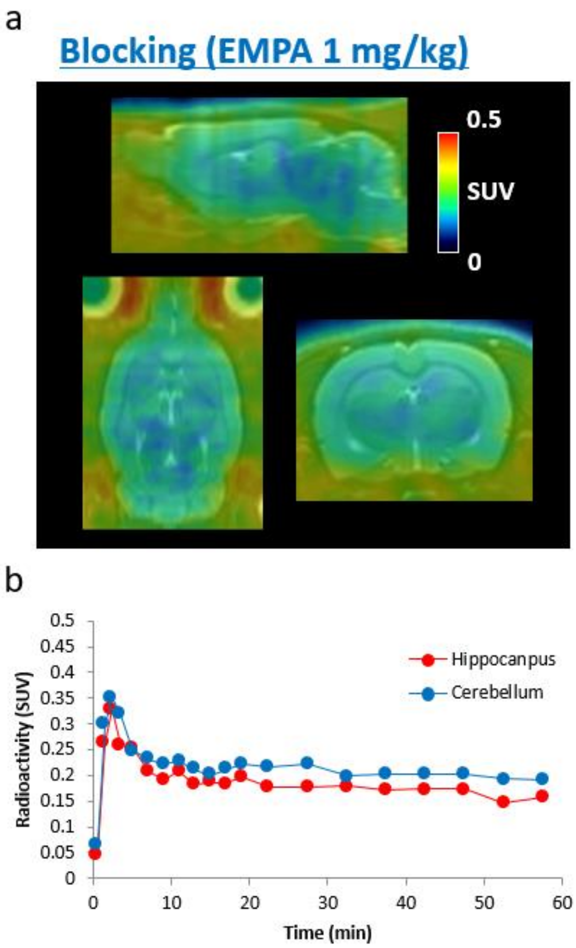

Figure S5. Representative PET images and time−activity curves of [^{11}C]**2** in blocking conditions (EMPA 1mg/kg) in rat brains. (a) Representative summed PET images (0−60 min) of [^{11}C]**2** in blocking conditions (Elacridar, 3 mg/kg); (b) TACs of [^{11}C]**2** in hippocampus and cerebellum.

2. Experimental Section

2.1 General information

All chemicals were ordered from commercial suppliers and used in the synthesis without further purification. The NMR spectra were collected on a 300 or 600 MHz spectrometer at room temperature. ^{1}H NMR chemical shifts were determined relative to internal $(CH_3)_4Si$ (TMS) at δ 0.00 ppm. ^{13}C NMR chemical shifts were determined relative to the signal of the solvent: $CDCl_3$ δ 77.16 ppm. Chemical shifts (δ) were reported in ppm, and coupling constants were reported in Hz. The multiplicities are abbreviated as follows: s

(singlet), d (doublet), t (triplet), m (multiplet), dd (doublet of doublets), and so forth. High-resolution mass data were recorded on a high-resolution mass spectrometer in the ESI mode. No promiscuity was observed in the assay of PAINS (Pan Assay Interference Compounds) for compounds **1**−**2** with two in silico filters (http://zinc15.docking.org/patterns/home and http://www.swissadme.ch/index.php).[1] High purities (≥95%) were also determined for compounds **1**−**2** by a reverse-phase HPLC (Agilent 1200). All animal studies were carried out in accordance with the institutional ethical guidelines. SD rats (male, 210−230 g, 7 weeks) were fed *ad libitum* with food and water under a 12 h light/12 h dark cycle. No unexpected or unusually high safety hazards were encountered.

2.2 Synthesis of antagonists **1-2** and labeling precursors **7** and **12**

Synthesis of *N*-(2-methoxyphenyl)-3-methylpyridine-2-sulfonamide (**4**)

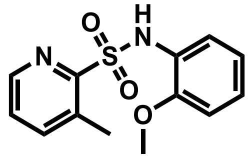

To a solution of 2-methoxy aniline **3** (3.6 g, 29.0 mol) in dry CH_2Cl_2 (50 mL), 3-methylpyridine-2-sulfonyl chloride (1.1 g, 6.0 mmol) was added dropwise at 0 ºC. Then the solution was stirred at 0 ºC for 2 h and stirred at room temperature for 1 h. After the reaction was completed (monitored by TLC), then water (20 mL) was added. The aqueous phase was extracted with CH_2Cl_2. The combined organic phase was washed with brine, dried over Na_2SO_4, and concentrated in a vacuum. The residue was purified by flash column chromatography on silica gel (PE/EA = 3/1) to give *N*-(2-methoxyphenyl)-3-methylpyridine-2-sulfonamide **4** (350.0 mg, 21% yield) as a brown oil. ^{1}H NMR (300 MHz, $CDCl_3$): δ 8.40 (s, 1H), 7.62–7.48 (m, 3H), 7.33–7.29 (m, 1H), 7.00 (t, J = 7.6 Hz, 1H), 6.85–6.79 (m, 2H), 3.80 (s, 3H), 2.63 (s, 3H). ^{13}C NMR (75 MHz, $CDCl_3$): δ 155.1, 149.1, 146.5, 144.6, 141.2, 133.7, 126.8, 124.8, 121.1, 119.9, 110.8, 56.0, 19.2.

Synthesis of methyl *N*-(2-methoxyphenyl)-*N*-((3-methylpyridin-2-yl)sulfonyl)glycinate (**5**)

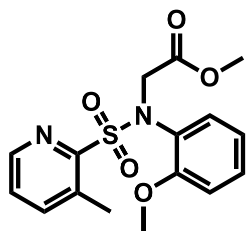

A solution of *N*-(2-methoxyphenyl)-3-methylpyridine-2-sulfonamide **4** (140.0 mg, 0.5 mmol), methyl 2-bromoacetate (91.0 mg, 0.6 mmol) and K_2CO_3 (110.0 mg, 0.8 mmol) in DMF (6 mL) was stirred at 25 °C for 12 h under N_2 atmosphere. After the reaction was completed (monitored by TLC), water (50 mL) was added, then the aqueous phase was extracted with ethyl acetate. The combined organic phase was washed with brine, dried over Na_2SO_4, and concentrated under reduced pressure. The residue was purified by flash column chromatography on silica gel (PE/EA=2/1) to give methyl *N*-(2-methoxyphenyl)-*N*-((3-methylpyridin-2-yl)sulfonyl)glycinate **5** (100.0 mg, 57% yield) as a yellow oil. ^{1}H NMR (300 MHz, $CDCl_3$): δ 8.60 (s, 1H), 7.70–7.61 (m, 2H), 7.41–7.32 (m, 2H), 7.00 (t, J = 6.4 Hz, 1H), 6.80 (d, J = 8.1 Hz, 1H), 4.68 (s, 2H), 3.74 (s, 3H), 3.44 (s, 3H), 2.33 (s, 3H). ^{13}C NMR (75 MHz, $CDCl_3$): δ 170.0, 156.2, 140.9, 133.9, 130.3, 126.9, 121.0, 111.3, 55.2, 52.2. HRMS (ESI): exact mass calcd for $C_{16}H_{19}N_2O_5S$ (M + H$^+$): 351.1009, found: 351.1018.

Synthesis of *N*-(2-methoxyphenyl)-*N*-((3-methylpyridin-2-yl)sulfonyl)glycine (**6**)

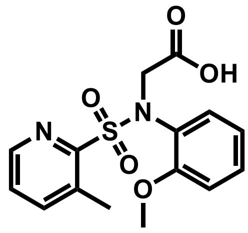

A solution of methyl *N*-(2-methoxyphenyl)-*N*-((3-methylpyridin-2-yl)sulfonyl)glycinate **5** (100.0 mg, 0.29 mmol) in 5 N NaOH/MeOH/THF (2 mL/2 mL/2 mL) was stirred at 60 °C for 12 h. After the reaction was completed (monitored by TLC), water (50 mL) was added, then the aqueous phase was extracted with ethyl acetate. Then the aqueous phase was

acidified by adding 1 N HCl to turn pH around 3 and extracted with ethyl acetate. The organic phase was washed with brine, dried over Na_2SO_4, and concentrated in a vacuum to give *N*-(2-methoxyphenyl)-*N*-((3-methylpyridin-2-yl)sulfonyl)glycine **6** (60.0 mg, 62% yield) as a red solid without further purification.

Synthesis of *N*-benzyl-*N*-ethyl-2-((N-(2-methoxyphenyl)-3-methylpyridine)-2-sulfonamido)acetamide (**1**)

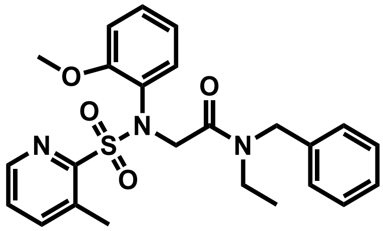

To a solution of *N*-(2-methoxyphenyl)-*N*-((3-methylpyridin-2-yl)sulfonyl)glycine **6** (60.0 mg, 0.18 mmol) in CH_2Cl_2 (6 mL) was added HATU (hexafluorophosphate azabenzotriazole tetramethyl uranium, 88.0 mg, 0.23 mmol) at 0 °C. Then the solution was stirred at 0 °C for 10 min. Then *N*-benzylethanamine **7** (48.0 mg, 0.36 mmol) and *N*,*N*-diisopropylethylamine (46.0 mg, 0.36 mmol) were added, and the mixture was stirred at room temperature for 12 h. After the reaction was completed (monitored by TLC), water (30 mL) was added, then the aqueous phase was extracted with ethyl acetate. The combined organic phase was washed with brine, dried over Na_2SO_4, and concentrated under reduced pressure. The residue was purified by flash column chromatography on silica gel (CH_2Cl_2/MeOH=100/0~10/1) to give *N*-benzyl-N-ethyl-2-((*N*-(2-methoxyphenyl)-3-methylpyridine)-2-sulfonamido)acetamide **1** (24.0 mg, 29% yield) as a white solid. HPLC purity: 96.4%. A mixture of two rotamers. ^{1}H NMR (600 MHz, $CDCl_3$) δ 8.54 [8.49] (d, *J* = 4.0 Hz, 1H), 7.64 [7.61] (dd, *J* = 7.8, 1.5 Hz, 1H), 7.58 [7.55] (d, *J* = 7.6 Hz, 1H), 7.36 - 7.21 (m, 5H), 7.15 [7.06] (d, *J* = 7.4 Hz, 2H), 6.93 (t, *J* = 7.6 Hz, 1H), 6.76 [6.73] (d, *J* = 8.2 Hz, 1H), 4.78 [4.70] (s, 2H), 4.67 [4.50] (s, 2H), 3.42 [3.31] (q, *J* = 7.1 Hz, 2H), 3.44 [3.43] (s, 3H), 2.27 [2.26] (s, 3H), 1.17 [0.97] (t, *J* = 7.1 Hz, 3H). ^{13}C NMR (151 MHz,

$CDCl_3$) δ 167.59, 167.55, 156.63, 156.58, 156.21, 146.54, 146.43, 141.07, 141.02, 137.48, 137.01, 133.81, 133.76, 133.68, 133.63, 130.11, 128.89, 128.54, 128.25, 127.55, 127.30, 126.64, 126.56, 126.27, 126.23, 120.94, 120.88, 111.38, 111.25, 55.22, 55.16, 52.63, 52.45, 50.19, 48.03, 41.21, 40.97, 19.76, 19.71, 13.80, 12.47. HRMS (ESI): exact mass calcd for $C_{24}H_{28}N_3O_4S$ ($M + H^+$): 454.1795, found: 454.1804.

Synthesis of *N*-benzyl-*N*-ethyl-2-((*N*-(2-hydroxyphenyl)-3-methylpyridine)-2-sulfonamido) acetamide (**7**)

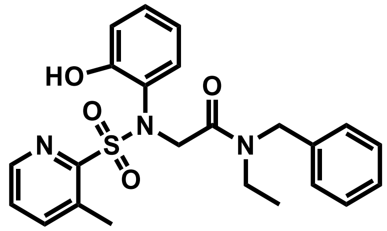

To a solution of *N*-benzyl-*N*-ethyl-2-((*N*-(2-methoxyphenyl)-3-methylpyridine)-2-sulfonamido) acetamide **1** (100.0 mg, 0.22 mmol) in CH_2Cl_2 (3 mL) was added BBr_3 (2.2 g, 8.80 mmol) at -10 °C. Then the mixture was stirred at room temperature for 5 min. After the reaction was completed (monitored by TLC), water (30 mL) was added, then the aqueous phase was extracted with CH_2Cl_2/MeOH (v/v = 10/1). The combined organic phase was washed with brine, dried over Na_2SO_4, and concentrated under reduced pressure. The residue was purified by silica gel plate (CH_2Cl_2/MeOH = 40/1) to give *N*-benzyl-*N*-ethyl-2-((N-(2-hydroxyphenyl)-3-methylpyridine)-2-sulfonamido)acetamide **7** (40.0 mg, 41% yield) as a white solid. HPLC purity: 99.6%. A mixture of two rotamers. ^{1}H NMR (600 MHz, $CDCl_3$) δ 8.54 [8.46] (d, *J* = 4.2 Hz, 1H), 7.60 [7.68] (d, *J* = 7.7 Hz, 1H), 7.45 [7.41] (dd, *J* = 7.7, 4.7 Hz, 1H), 7.32 -7.14 (m, 6H), 7.14 (d, *J* = 7.3 Hz, 1H), 7.03 – 6.98 (m, 1H), 6.75 [6.72] (td, *J* = 7.6, 1.1 Hz, 1H), 5.16 [4.63] (s, 2H), 4.49 [4.27] (s, 2H), 3.44 [3.23] (s, 2H), 2.54 [2.53] (s, 3H), 1.13 [1.10] (t, *J* = 7.2 Hz, 3H). ^{13}C NMR (151 MHz, $CDCl_3$) δ 169.59, 169.54, 156.49, 156.37, 155.83, 155.71, 145.64, 145.51, 142.14, 136.79, 135.79, 134.62, 134.56, 131.18, 131.12, 130.90, 130.83, 129.16, 128.78, 128.28, 127.97,

127.67, 126.94, 126.90, 126.85, 126.56, 119.94, 119.93, 118.57, 118.51, 77.37, 77.16, 76.95, 54.72, 54.60, 50.08, 48.93, 42.05, 40.97, 19.22, 19.18, 13.64, 12.54. LRMS (ESI): $C_{24}H_{28}N_3O_4S$ (M + H^+): 440.2, found: 440.2. HRMS (ESI): exact mass calcd for $C_{23}H_{26}N_3O_4S$ (M + H^+): 440.1639, found: 440.1626.

Synthesis of *N*-(6-methoxypyridin-3-yl)-3-methylpyridine-2-sulfonamide (**9**)

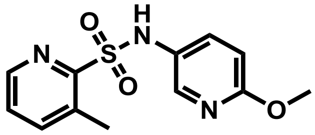

To a solution of 3-methylpyridine-2-sulfonamide **8** (500.0 mg, 2.91 mmol) in dry DMF (15 mL), Cs_2CO_3 (1.9 g, 5.8 mmol), CuI (276.2 mg, 1.43 mmol), and 5-iodo-2-methoxypyridine (1.0 g, 4.26 mmol) were added under N_2 atmosphere. The mixture was treated with microwave at 150 ºC for 30 min. Then the mixture was cooled to room temperature, and water (100 mL) was added. Then the aqueous phase was extracted with CH_2Cl_2. The combined organic layer was washed with water and brine, dried over Na_2SO_4, and concentrated under reduced pressure to get the crude, which was purified by flash column chromatography on silica gel (0~10% MeOH in CH_2Cl_2) to give *N*-(6-methoxypyridin-3-yl)-3-methylpyridine-2-sulfonamide **9** (490.0 mg, 60% yield) as a light green solid. ^{1}H NMR (300 MHz, $CDCl_3$): δ 8.48 (d, J = 4.3 Hz, 1H), 8.14 (d, J = 2.6 Hz, 1H), 7.68–7.62 (m, 2H), 7.56 (s, 1H), 7.36 (dd, J = 7.7 Hz, J = 4.7 Hz, 1H), 6.66 (d, J = 9.0 Hz, 1H), 3.88 (s, 3H), 2.59 (s, 3H). ^{13}C NMR (75 MHz, $CDCl_3$): δ 162.4, 155.1, 146.3, 143.3, 141.6, 136.9, 133.9, 127.2, 126.9, 111.1, 54.0, 19.0.

Synthesis of methyl *N*-(6-methoxypyridin-3-yl)-*N*-((3-methylpyridin-2-yl)sulfonyl)glycinate (**10**)

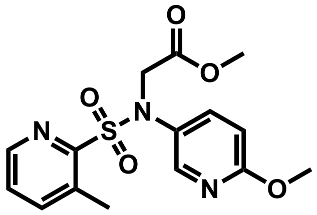

To a solution of *N*-(6-methoxypyridin-3-yl)-3-methylpyridine-2-sulfonamide (686 mg, 2.46 mmol) in DMSO (25 mL), *t*BuOK (360.0 mg, 3.2 mmol) and methyl 2-bromoacetate (455.4 mg, 3.0 mmol) were added. The mixture was stirred at 60 °C for 12 h. The mixture was diluted with ethyl acetate, washed with water and brine, dried over Na_2SO_4, and concentrated under reduced pressure to give methyl *N*-(6-methoxypyridin-3-yl)-*N*-((3-methylpyridin-2-yl)sulfonyl)glycinate **10** (860.0 mg, 99% yield) without further purification. ^{1}H NMR (300 MHz, $CDCl_3$): δ 8.49 (d, J = 4.5 Hz, 1H), 8.38 (d, J = 2.5 Hz, 1H), 7.87 (dd, J = 8.7 Hz, J = 2.5 Hz, 1H), 7.66 (d, J = 7.8 Hz, 1H), 7.38 (dd, J = 7.8 Hz, J = 4.7 Hz, 1H), 6.71 (d, J = 8.9 Hz, 1H), 4.69 (s, 2H), 3.90 (s, 3H), 3.72 (s, 3H), 2.56 (s, 3H). ^{13}C NMR (75 MHz, $CDCl_3$): δ 169.3, 163.3, 156.1, 147.9, 145.9, 141.5, 141.1, 133.8, 130.5, 126.7, 111.2, 54.8, 54.1, 52.5, 18.8.

Synthesis of *N*-(6-methoxypyridin-3-yl)-*N*-((3-methylpyridin-2-yl)sulfonyl)glycine (**11**)

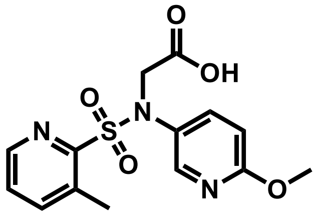

To a solution of methyl *N*-(6-methoxypyridin-3-yl)-*N*-((3-methylpyridin-2-yl)sulfonyl)glycinate **10** (860.0 mg, 2.45 mmol) in MeOH (25 mL), NaOH (aq, 2 N, 12 mL) was added. The mixture was stirred at 60 ºC for 3 h. Then the pH of the mixture was adjusted to around 6 by adding HCl (aq. 2 N). The mixture was extracted with ethyl acetate. The combined organic layer was washed with water and brine, dried over Na_2SO_4, and concentrated under reduced pressure to give *N*-(6-methoxypyridin-3-yl)-*N*-((3-

methylpyridin-2-yl)sulfonyl)glycine **11** (700.0 mg, 85% yield) as a pink solid.

Synthesis of *N*-benzyl-*N*-ethyl-2-((*N*-(6-methoxypyridin-3-yl)-3-methylpyridine)-2-sulfonamido)acetamide (**2**)

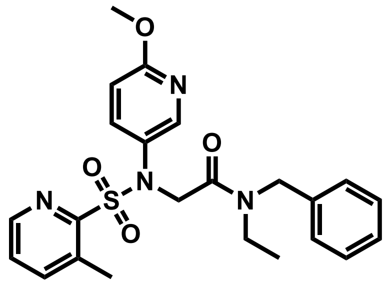

To a solution of *N*-(6-methoxypyridin-3-yl)-*N*-((3-methylpyridin-2-yl)sulfonyl)glycine **11** (500.0 mg, 1.48 mmol) in dry CH_2Cl_2 (25 mL), HATU (hexafluorophosphate azabenzotriazole tetramethyl uranium, 1.1 g, 2.89 mmol) was added, and the mixture was stirred at room temperature for 10 min. *N*,*N*-Diisopropylethylamine (478.4 mg, 3.7 mmol), and *N*-benzylethanamine 7 (400.0 mg, 2.96 mmol) were added to the above solution, and the mixture was stirred at room temperature for 12 h. Then the mixture was quenched with water and extracted with CH_2Cl_2. The combined organic layer was washed with water and brine, and concentrated under reduced pressure to get the crude, which was purified by flash column chromatography on silica gel to give *N*-benzyl-*N*-ethyl-2-((*N*-(6-methoxypyridin-3-yl)-3-methyl-pyridine)-2-sulfonamido)acetamide **2** (270.0 mg, 40% yield) as an orange oil. HPLC purity: 99.4%. A mixture of two rotamers. ^{1}H NMR (600 MHz, $CDCl_3$) δ 8.49 [8.41] (d, *J* = 4.3 Hz, 1H), 8.37 [8.31] (d, *J* = 2.3 Hz, 1H), 7.96 [7.93] (dd, *J* = 8.9, 2.4 Hz, 1H), 7.65 [7.62] (d, *J* = 7.7 Hz, 1H), 7.39 - 7.32 (m, 2H), 7.29 - 7.22 (m, 2H), 7.16 [7.09] (d, *J* = 7.5 Hz, 2H), 6.72 [6.71] (d, *J* = 8.9 Hz, 1H), 4.88 [4.80] (s, 2H), 4.60 [4.53] (s, 2H), 3.93 [3.92] (s, 3H), 3.36 (m, 2H), 2.54 [2.52] (m, 3H), 1.17 [1.01] (t, *J* = 7.1 Hz, 3H). ^{13}C NMR (151 MHz, $CDCl_3$) δ 167.09, 167.03, 162.88, 156.15, 156.13, 147.13, 147.07, 145.94, 145.85, 141.91, 141.82, 141.47, 141.40, 137.18, 136.52, 133.82, 133.76, 130.40, 129.08, 128.65, 128.12, 127.84, 127.49, 126.72, 126.65, 126.55, 110.99, 110.92, 77.37, 77.16, 76.95, 55.01, 54.87, 54.34, 50.09, 48.14, 41.41, 40.98, 18.86, 18.85,

13.85, 12.57. LRMS (ESI): $C_{23}H_{27}N_4O_4S$ (M + H^+): 455.2, found: 455.2. HRMS (ESI): exact mass calcd for $C_{23}H_{27}N_4O_4S$ (M + H^+): 455.1748, found: 455.1737.

Synthesis of *N*-benzyl-*N*-ethyl-2-((*N*-(6-hydroxypyridin-3-yl)-3-methylpyridine)-2-sulfonamido)acetamide (**12**)

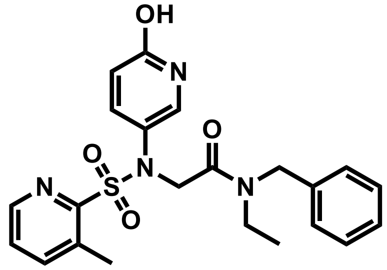

The compound *N*-benzyl-*N*-ethyl-2-((*N*-(6-methoxypyridin-3-yl)-3-methylpyridine)-2-sulfonamido)acetamide **2** (200.0 mg, 0.44 mmol) was dissolved in HBr/HOAc (15 mL) and the mixture was stirred at 60 °C for 12 h. The pH of the mixture was adjusted to 5-6 with NaOH (aq.), and the mixture was extracted with CH_2Cl_2. The combined organic layer was washed with water and brine, dried over Na_2SO_4, and concentrated under reduced pressure to get the crude, which was purified by flash column chromatography on silica gel to give *N*-benzyl-*N*-ethyl-2-((*N*-(6-hydroxypyridin-3-yl)-3-methylpyridine)-2-sulfonamido)acetamide **12** (87.2 mg, 45% yield) as a purple solid. HPLC purity: 97.7%. A mixture of two rotamers. ^{1}H NMR (600 MHz, $CDCl_3$) δ 8.46 [8.37] (d, *J* = 4.1 Hz, 1H), 7.81 [7.75] (dd, *J* = 9.7, 2.7 Hz, 1H), 7.74 [7.67] (d, *J* = 2.6 Hz, 1H), 7.66 [7.62] (d, *J* = 7.6 Hz, 1H), 7.39 – 7.31 (m, 2H), 7.27 – 7.19 (m, 2H), 7.13 (d, *J* = 7.5 Hz, 2H), 6.43 (t, *J* = 8.4 Hz, 1H), 4.73 [4.64] (s, 2H), 4.532 [4.526] (s, 2H), 3.37 [3.29] (q, *J* = 7.1 Hz, 2H), 2.57 [2.54] (s, 3H), 1.14 [1.04] (t, *J* = 7.1 Hz, 3H). ^{13}C NMR (151 MHz, $CDCl_3$) δ 167.14, 167.05, 164.29, 155.89, 155.85, 145.93, 145.84, 144.76, 144.69, 141.56, 141.49, 137.09, 137.05, 136.34, 133.80, 133.74, 129.08, 128.66, 128.09, 127.86, 127.53, 126.81, 126.73, 126.38, 121.47, 119.55, 119.48, 54.68, 54.57, 50.03, 48.22, 41.62, 40.92, 18.79, 18.77, 13.80, 12.61. LRMS (ESI): $C_{22}H_{25}N_4O_4S$ (M + H^+): 441.2, found: 441.2. HRMS (ESI): exact mass calcd for $C_{22}H_{25}N_4O_4S$ (M + H^+): 441.1591, found: 441.1600.

2.3 *Human OX_2 receptor binding (agonist radioligand) assays and CYP enzymes and hERG inhibition assays*

Human OX_2 receptor binding (agonist radioligand) assays were finished by Gifford Bioscience Limited. Competition binding assays were carried out in 96-well polypropylene plates in a final volume of 250 μL per well. To each well were added 150 μL Human OX_2 Receptor Membranes (MultiSpan Inc, MC1034), 50 μL of non-specific or test compound or buffer (50 mM Tris, 5 mM $MgCl_2$, 0.1 mM EDTA, pH 7.4), and 50 μL radioligand ([^{3}H]EMPA) solution in the buffer. The plate was incubated at 30 °C for 60 minutes with gentle agitation. The incubation was stopped by vacuum filtration onto presoaked (Tris buffer with PEI) GF/C filters using a 96-well FilterMate™ harvester, followed by 5 washes with ice-cold wash buffer (50 mM Tris, 5 mM $MgCl_2$, 0.1 mM EDTA, pH 7.4). Filters were then dried under a warm air stream and sealed in polyethylene, a scintillation cocktail was added, and the radioactivity was counted in a Wallac® TriLux 1450 MicroBeta counter.

For each drug concentration, non-specific binding was subtracted from total binding to give specific binding. Data were fitted using the non-linear curve fitting routines in Prism® (Graphpad Software Inc) to determine IC_{50}. K_i was subsequently calculated using the Cheng-Prusoff equation.

CYP enzymes and hERG inhibition assays were finished by Reaction Biology Corporation.

2.4 *Radiolabeling of [^{11}C]**1** and [^{11}C]**2***

According to the previous reports, the procedure for [^{11}C]CH_3I formation was modified in this work.[2-4] In brief, [^{11}C]CH_3I was yielded from cyclotron-produced [^{11}C]CO_2 by ^{14}N(p, α)^{11}C nuclear reaction. In this study, [^{11}C]CO_2 from a cyclotron was bubbled into the 0.4 M solution of $LiAlH_4$ in THF (300 μL). After evaporation, the remaining reaction mixture added 57% of hydroiodic acid aqueous solution (300 μL). With heating, the resulting [^{11}C]CH_3I was then transferred under helium gas into a precooled (−15 to −20 °C) reaction vessel containing the precursor **7** or **12** (1 mg), NaOH (0.5 M, 5 μL for [^{11}C]**1**), or Cs_2CO_3

(6.6 mg for [^{11}C]**2**), and anhydrous DMF (0.3 mL). When the radioactivity of this mixture reached a plateau, remove the transfer needles from the vessel cap. The reaction was heated to 80 °C and maintained for 5 min. After the reaction completed, the mixture was neutralized by a buffer solution and this mixture was loaded into a preparative HPLC column. HPLC purification was completed on a CAPCELL PAK C_{18} column (10 mm ID X 250 mm) using the mobile phase of CH_3CN/H_2O (60/40, v/v) at a flow rate of 5 mL/min. The fractions corresponding to [^{11}C]**1** (8.1 min) and [^{11}C]**2** (8.0 min) was collected into a flask containing Tween 80 (75 µL) and ethanol (150 µL), evaporated to dryness *in vacuo*, re-dissolved in 5 mL of sterile normal saline and passed through a 0.22 µm Millipore filter for analysis and application experiments. The synthesis time was 40 min. The radiochemical yields (RCY, decay-corrected) were 71% for [^{11}C]**1** and 20% for [^{11}C]**2** based on [^{11}C]CO_2 with > 99% radiochemical purity. The molar activities of [^{11}C]**1** and [^{11}C]**2** were 210 GBq/µmol and 185 GBq/µmol at the end of synthesis (EOS), respectively.

The specific activities of [^{11}C]**1** and [^{11}C]**2** were calculated based on mass calibration curves.

Calibration curve of compound **1**

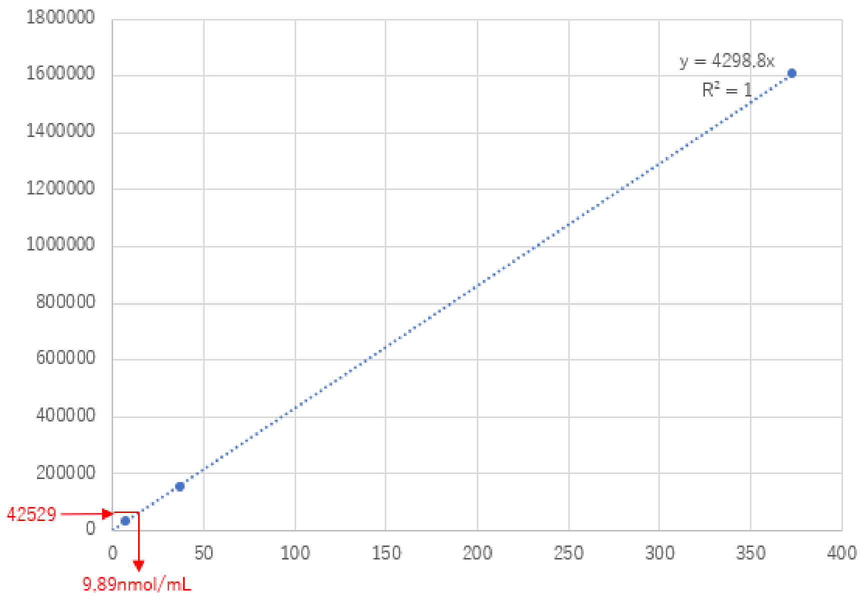

The specific activity of [^{11}C]**1** was calculated to be 5.68 Ci/μmol (210 GBq/μmol).

Calibration curve of compound **2**

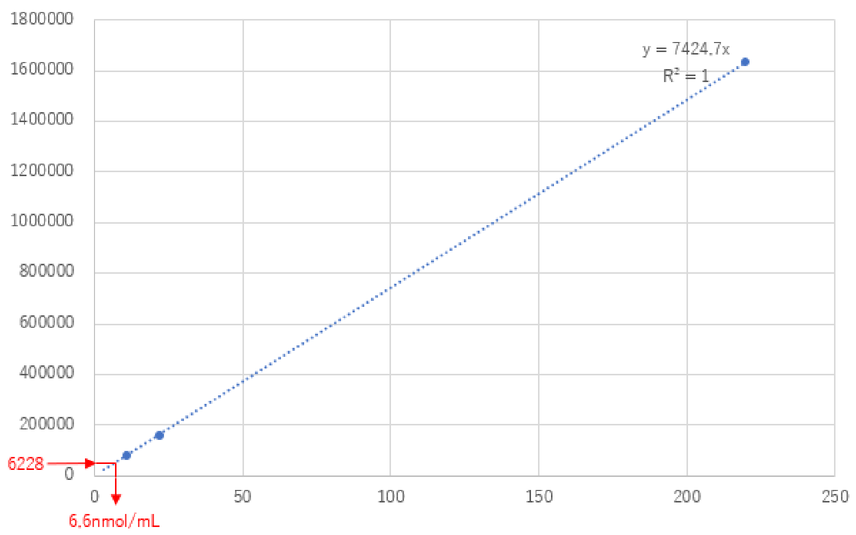

The specific activity of [^{11}C]**2** was calculated to be 5 Ci/μmol (185 GBq/μmol).

2.5 In vitro autoradiography

The method for in vitro autoradiography was slightly modified from previous reports.[5] Rat brain was cut into 20 μm sections, stored at −80 °C, and ready for the experiment. The rat brain sections were preincubated with Tris−HCl buffer (50 mM) containing $MgCl_2$ (2 mM) and $CaCl_2$ (1.2 mM) for 20 min at ambient temperature, followed by incubation with [^{11}C]**1** or [^{11}C]**2**. For blocking studies, compound **1** (10 μM), **2** (10 μM), or EMPA (10 μM) was added to incubation solution. Then the brain sections were washed with ice-cold buffer for three times and dipped in cold distilled water for 10 s. The brain sections were dried with cold air. The exposure time for the phosphor plate in the autoradiography study is 1 h.

2.6 Small-animal PET imaging studies

The imaging studies were conducted according to the previous literature[6,7] with minor modifications. PET scans were carried out by an Inveon PET scanner. Sprague-Dawley rats were kept under anesthesia with 1-2% (v/v) isoflurane during the scan. The radiotracer [^{11}C]**1** (37–42 MBq) or [^{11}C]**2** (32–37 MBq) was injected *via* a preinstalled catheter into the tail vein. A dynamic scan in 3D mode was acquired for 60 min. For blocking studies, a solution of elacridar (3 mg/kg) in 300 µL saline containing 10% DMSO, 40% polyethylene glycol, and 5% Tween® 80 was injected at 20 min *via* the pre-embedded tail vein catheter before PET tracer injection. An MR template of SD rats was applied for PET/MR merged images. The radioactivity was decay-corrected and expressed as the standardized uptake value (SUV). SUV = (radioactivity per mL tissue / injected radioactivity) x body weight.

*2.7 HPLC radio-chromatograms of [^{11}C]**1** and [^{11}C]**2***

Semi-prep radio-HPLC chromatogram of [^{11}C]**1**

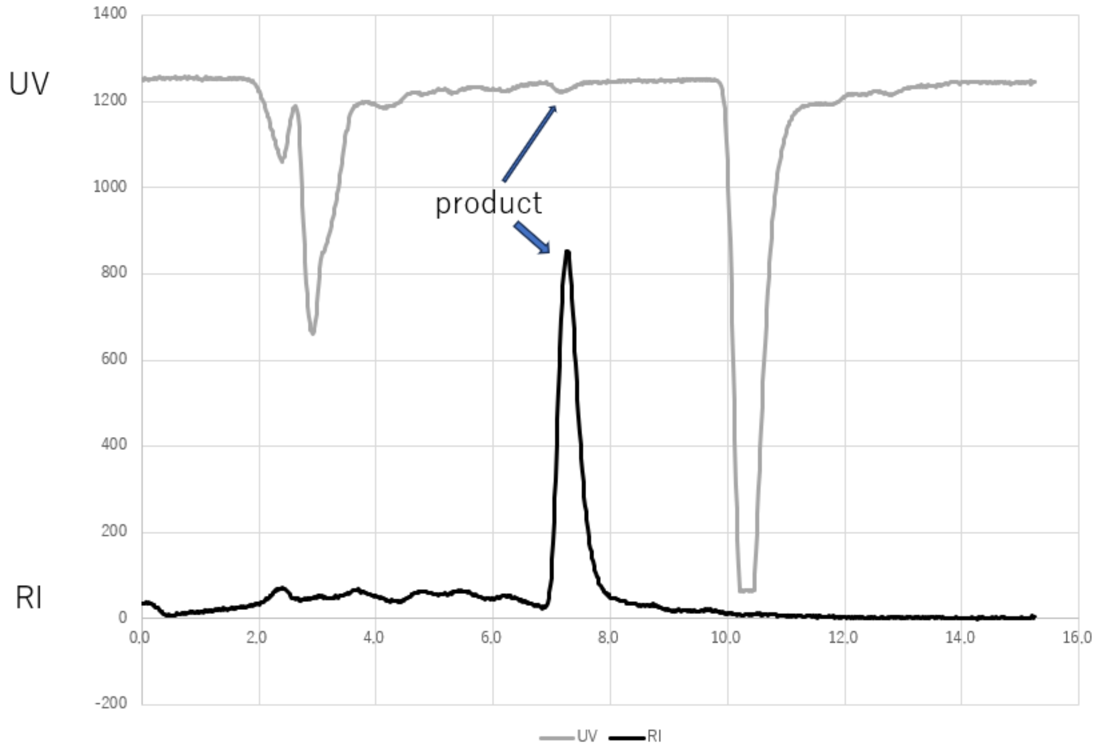

Analytical radio-HPLC chromatogram of pure [^{11}C]**1**

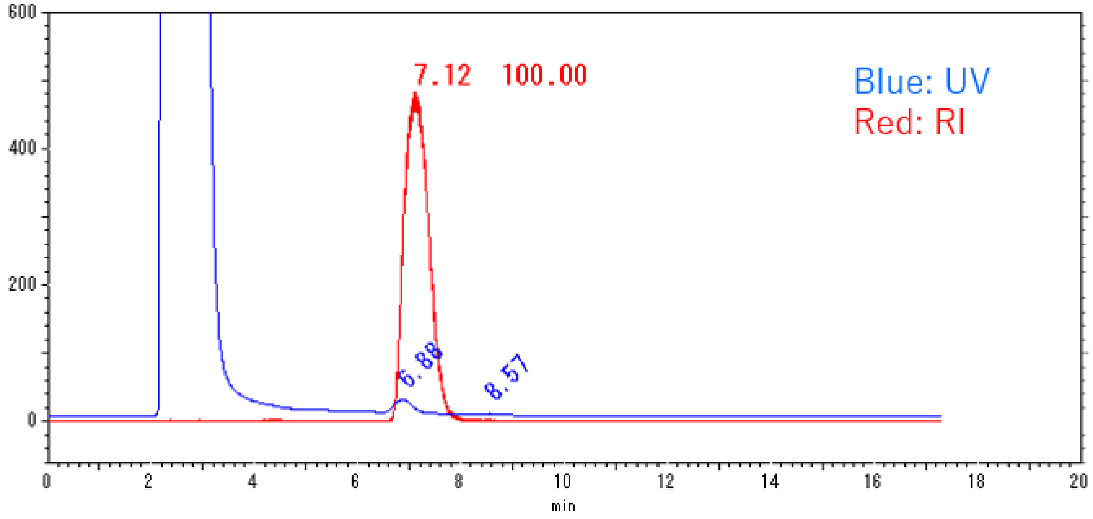

Analytical radio-HPLC chromatogram of co-injection of [^{11}C]**1** with compound **1**

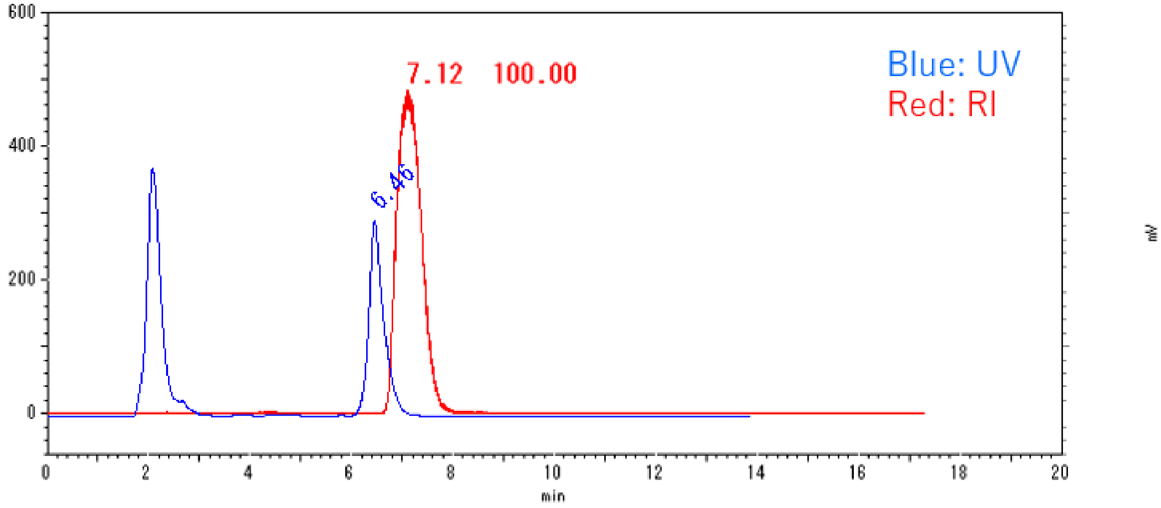

Semi-prep radio-HPLC chromatogram of [^{11}C]**2**

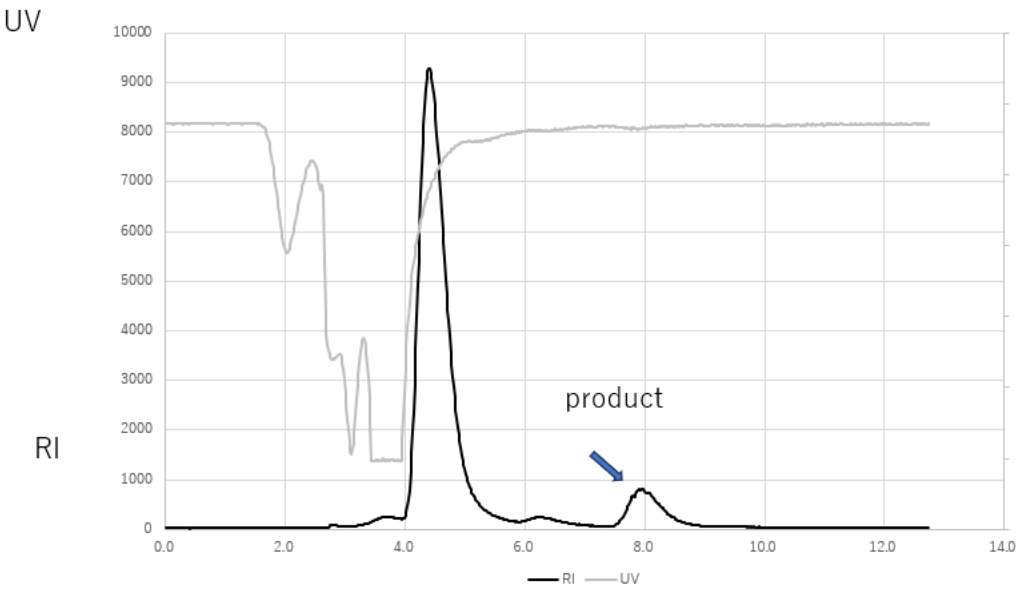

Analytical radio-HPLC chromatogram of pure [^{11}C]**2**

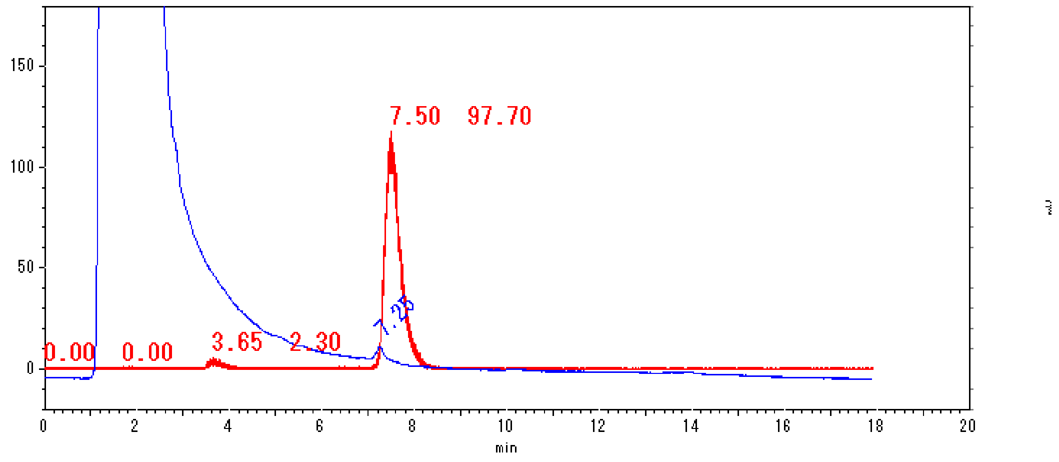

Analytical radio-HPLC chromatogram of co-injection of [^{11}C]**2** with compound **2**

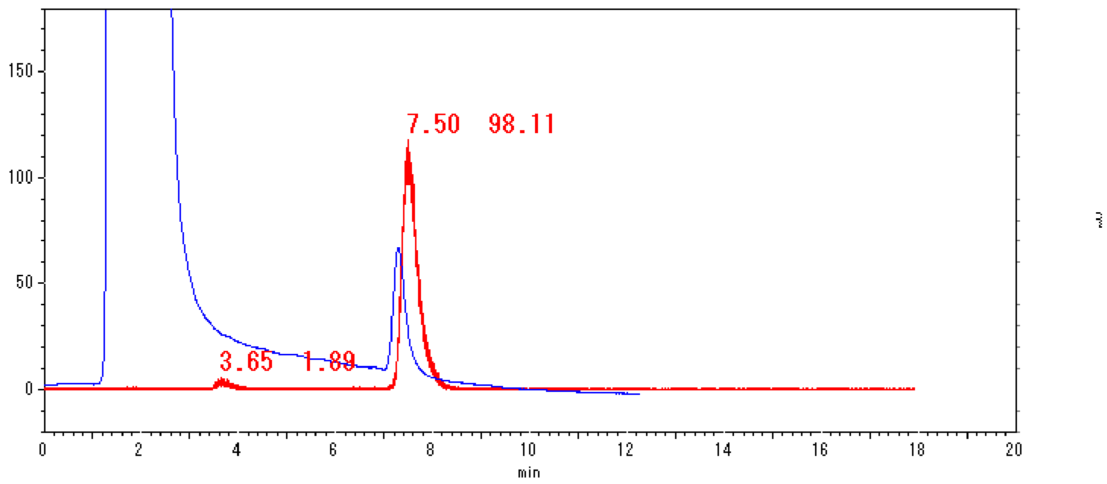

*2.8 NMR spectra of key compounds **1** and **2***

^{1}H NMR spectra of compound **1** ($CDCl_3$, 600 MHz)

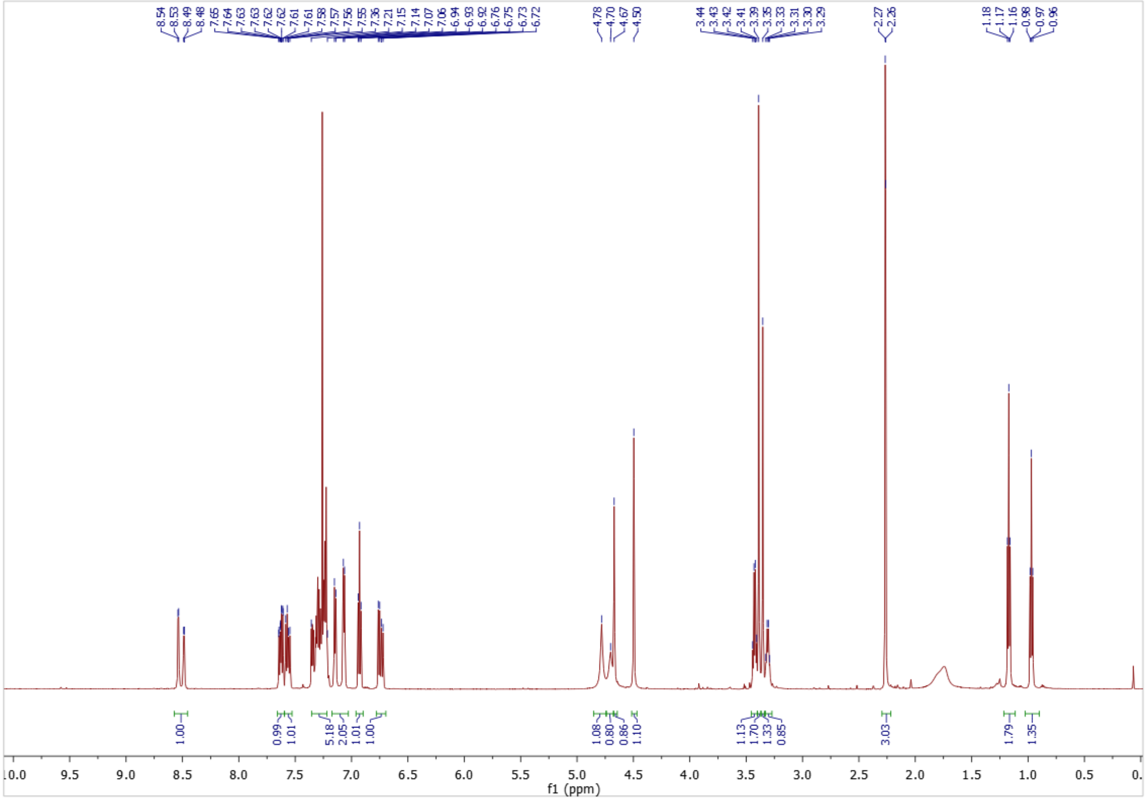

^{13}C NMR spectra of compound **1** ($CDCl_3$, 151 MHz)

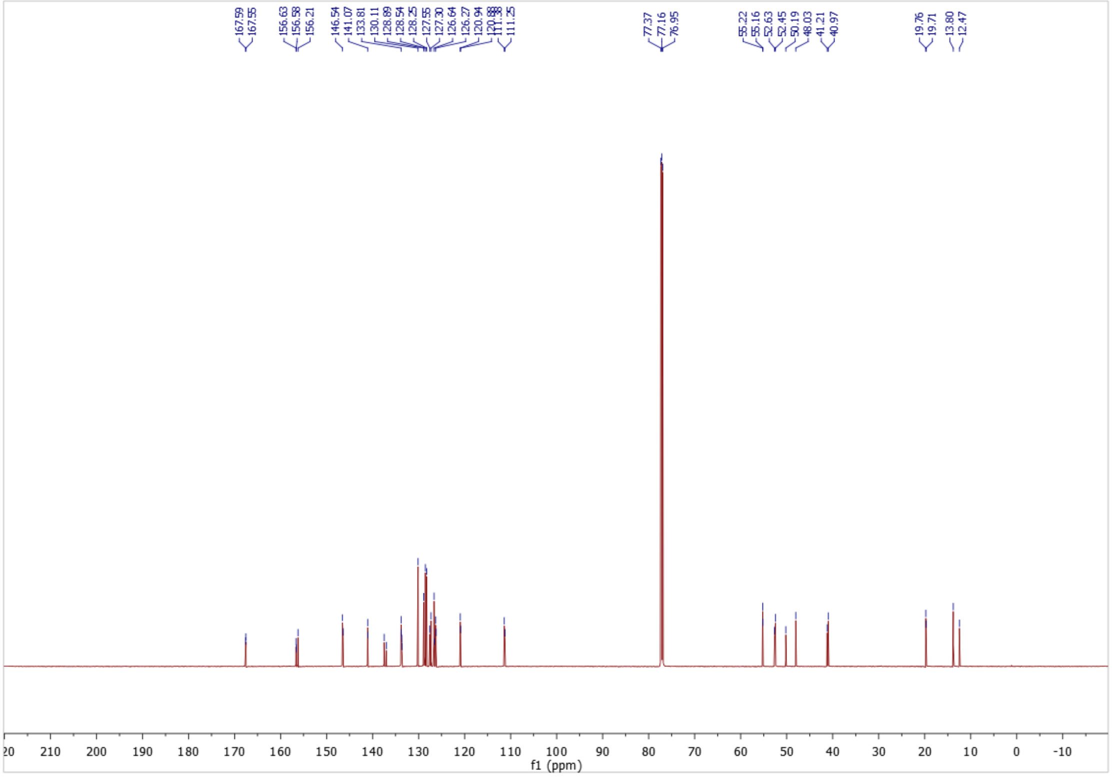

^{1}H NMR spectra of compound **2** ($CDCl_3$, 600 MHz)

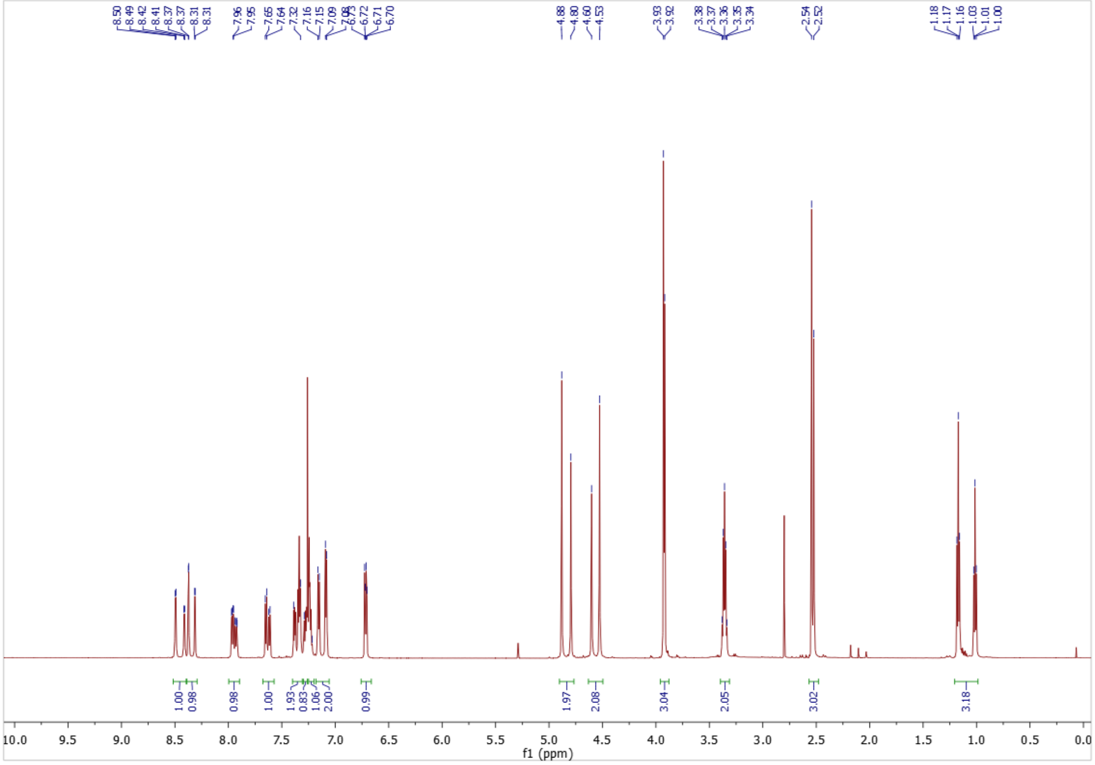

^{13}C NMR spectra of compound **2** ($CDCl_3$, 151 MHz)

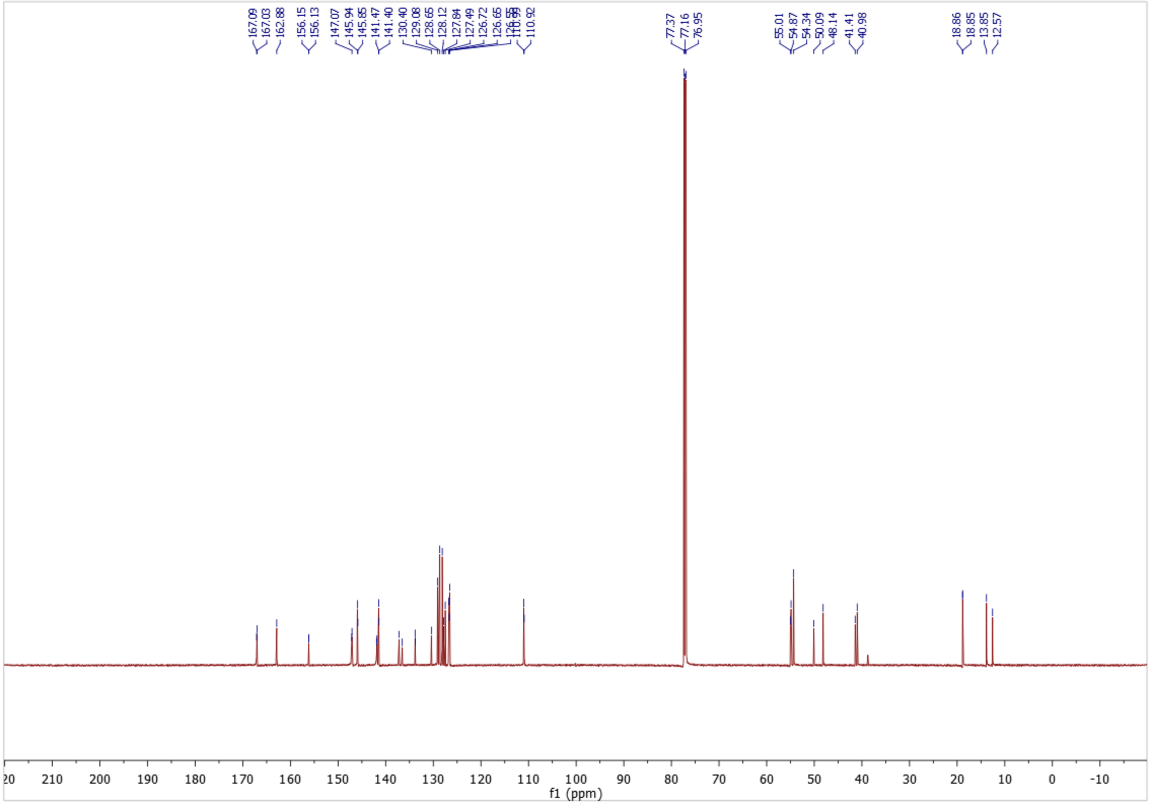

References

1. Baell, J. B.; Holloway, G. A. *J. Med. Chem.* **2010**, *53*, 2719−2740.
2. Deng, X.; Rong, J.; Wang, L.; Vasdev, N.; Zhang, L.; Josephson, L.; Liang, S. H. *Angew. Chem., Int. Ed.* **2019**, *58*, 2580−2605.
3. Huang, Y.; Narendran, R.; Bischoff, F.; Guo, N.; Zhu, Z.; Bae, S.-A.; Lesage, A. S.; Laruelle, M. *J. Med. Chem.* **2005**, *48*, 5096−5099.
4. Shimoda, Y.; Yamasaki, T.; Fujinaga, M.; Ogawa, M.; Kurihara, Y.; Nengaki, N.; Kumata, K.; Yui, J.; Hatori, A.; Xie, L.; Zhang, Y.; Kawamura, K.; Zhang, M.-R. *J. Med. Chem.* **2016**, *59*, 3980−3990.
5. Chen, Z.; Mori, W.; Fu, H.; Schafroth, M. A.; Hatori, A.; Shao, T.; Zhang, G.; Van, R. S.; Zhang, Y.; Hu, K.; Fujinaga, M.; Wang, L.; Belov, V.; Ogasawara, D.; Giffenig, P.; Deng, X.; Rong, J.; Yu, Q.; Zhang, X.; Papisov, M. I.; Shao, Y.;

Collier, T. L.; Ma, J.-A.; Cravatt, B. F.; Josephson, L.; Zhang, M.-R.; Liang, S. H. *J. Med. Chem.* **2019**, *62*, 8866−8872.

6. Wang, L.; Mori, W.; Cheng, R.; Yui, J.; Hatori, A.; Ma, L.; Zhang, Y.; Rotstein, B. H.; Fujinaga, M.; Shimoda, Y.; Yamasaki, T.; Xie, L.; Nagai, Y.; Minamimoto, T.; Higuchi, M.; Vasdev, N.; Zhang, M.-R.; Liang, S. H. *Theranostics* **2016**, *6*, 1145−1159.
7. Cheng, R.; Mori, W.; Ma, L.; Alhouayek, M.; Hatori, A.; Zhang, Y.; Ogasawara, D.; Yuan, G.; Chen, Z.; Zhang, X.; Shi, H.; Yamasaki, T.; Xie, L.; Kumata, K.; Fujinaga, M.; Nagai, Y.; Minamimoto, T.; Svensson, M.; Wang, L.; Du, Y.; Ondrechen, M. J.; Vasdev, N.; Cravatt, B. F.; Fowler, C.; Zhang, M.-R.; Liang, S. H. *J. Med. Chem.* **2018**, *61*, 2278−2291.
